# On the Extensive Diffusion and Frequency of Starch Corpuscles in the Tissues of the Human Body

**Published:** 1855-09

**Authors:** Thomas Albert Carter


*On the Extensive Diffusion and Frequency of Starch Corpuscles in the Tissues of the Human Body.* By THOMAS ALBERT CARTER.—Two years have now almost elapsed since Virchow first published his paper upon the presence of cellulose in the brain and spinal cord of man; in which he proved that the ependyma and its continuations contained bodies which, with sulphuric acid and iodine, struck a violet color, concluding from this circumstance that their chemical composition was that of cellulose. He also found them in the olfactory and optic nerves, and in a "waxy" spleen. Subsequently, Rokitansky observed similar bodies in the optic nerve, and Kölliker in the retina. It remained, however, for Mr. Busk to establish the fact, that the so-called cellulose corpuscles of Virchow were absolutely identical in every property—whether optical, physical, or chemical—with starch.* He, moreover, showed that this substance occupies a place in the tubular and vesicular portions of the human cerebrum and cerebellum. Since the valuable observations of Busk made their appearance, Mr. Stratford, of Toronto, has published a short paper† "upon the presence of starch in the blood of an epileptic patient," in which he also states, that he has met with it in the eye of a boy, removed in consequence of fungus hæmatodes, and in several specimens of urine.

In December last, an unsuccessful attempt to make out the intimate structure of an indurated tumour involving the optic nerve, induced me, as a last resource, to apply the appropriate tests for the detection of

* *Quarterly Journal of Microscopical Science*, for January, 1854.
† Ibid., for January, 1855.

cellulose. To my great surprise, after the addition of the reagents, I noticed a number of bodies, generally circular or oval in outline, passing from the lightest shades of purple to the densest black: these were evidently the cellulose corpuscles of Virchow, or the starch bodies of Busk, the latter of which they have since proved to be. Shortly afterwards I observed the same bodies in the pineal gland of man, and of the sheep. I then abandoned this subject, more urgent duties soliciting my attention, but resumed its investigation about the middle of last May, and have continued it up to the present time.

It is unnecessary to give a description of the "amyloid" corpuscles, and the proofs in favor of their being actually starch, for this would be simply reiterating the statements of Busk respecting them, which by experiment I have found to be perfectly correct. It may, however, be remarked that, in my examinations of structures from the human subject, I have encountered two distinct varieties—the one (described by Busk) resembling wheat starch, the other, and rarer kind, corresponding in every particular with that derived from the potato. In dimensions they vary much, ranging from 1–195th to less than a 1–4000th of an inch in diameter, the usual size being the 1–1000th.

The following are the observations I have had an opportunity of making on a number of cases, and these, it must be remembered, have not been selected, but given in the order in which they presented themselves, none being suppressed.

*Observation* 1. A man, N., æt. 39 years who died of phthisis pulmonalis, complicated with albuminuria.

A micro-chemical examination of the liver, which was of twice its ordinary weight, and presented in a marked degree the characters of waxy degeneration, revealed the presence of starch in great abundance. One demonstration exhibited fifty-two bodies of the ordinary size, adhering to, or imbedded in, a mass of transparent homogeneous tissue.

In the spleen and kidney, which were also "waxy," starch was found, abundantly in the former, in the latter somewhat sparingly.

*Obs.* 2. A woman, J. B., æt. 59 years, who died of apoplexy.

The tubular matter of the cerebrum, immediately surrounding the appoplectic clot, was subjected to an examination, and in it were found, associated with numerous compound granular cells, numbers of starch corpuscles. This observation does not apply to the healthy portions of the same brain, for, after a prolonged search, none could be detected. An ordinary amount* was observed in the spleen, kidney, and liver of this subject, all of which appeared perfectly mormal.

*Obs.* 3. A female, M. R., æt. 24, who died of phthisis pulmonalis.

* The expressions "small quantity," etc., I am aware, are extremely vague and indefinite; yet, by attaching to them a certain numerical value, they communicate an idea of amount with sufficient exactitude, and obviate the necessity of giving the precise number of bodies in each demonstration, which, to say the least of it, would have been a very tedious process. By the term "small quantity," as applied to the contents of a preparation, I wish the reader to understand from one to three corpuscles; by "ordinary amount," from five to eight; by "large amount," from ten to twenty or more. The value of synonyms, or intermediate expressions, will, of course, be duly appreciated.

In the liver, which was somewhat waxy and slightly enlarged were found starch bodies of irregular form, as if developed in masses under pressure.

The spleen yielded a few. This, like the former organ, was in some degree waxy.

They were very numerous, and of large dimensions, in the kidney many attaining to the 1–400th part of an inch in diameter.

I noticed, in the pancreas and mesenteric glands of this subject, numbers of corpuscles, generally of oval form, measuring about the 1–400th of an inch in their longest, and about half that in their shortest diameter. The hilum was placed near one extremity, with a series of concentric elipsoidal markings surrounding it. Moreover, when seen by polarised light, they exhibited the four black or colored radii diverging from the hilum; in fact, they were in every respect identical with the amylum of the potato.

*Obs.* 4. A female, J. H., æt. 23 years, who died of phthisis pulmonalis.

Starch was found in the liver, kidney, spleen, pancreas, mesenteric glands, and supra-renal capsules, all of which organs were healthy, with the exception of the mesenteric glands, these being slightly enlarged: also in an indurated tubercular mass from the lung.

*Obs.* 5. A man, J. B., æt. 38 years. The immediate cause of death unknown. The heart and larger vessels, however, were diseased.

In the liver (slightly enlarged, but in other respects healthy,) kidneys, supra-renal capsules, spleen, corpora striata, pituitary body, and Pacchionian glands, starch was found. None could be discovered in the pancreas, after a very careful and prolonged search. This organ was very flaccid, and apparently diseased.

*Obs.* 6. A man, Y., æt. 17 years, who died of general anasarca.

The liver of this subject was congested, and to a small extent fatty; the spleen rather pulpy; both exhibited the usual amount. The pancreas, which was flabby, yielded a very small quantity. In a mass of degenerate pus found adhering to the peritoneal surface of the bladder, and in chronic pericardial exudation matter, they were also present.

*Obs.* 7. A boy, P., æt. 14 years, who died of apoplexy.

The liver, spleen, kidneys, supra-renal capsules, thyroid body, tonsils, and brain, contained starch in greater or less quantity. The kidney, in this case, was the viscus in which they were principally developed, both as regards number and size. By mensuration, I ascertained that one of them exceeded the 1–200th of an inch linear. All the organs appeared quite healthy.

*Obs.* 8. A youth, æt. 17 years, whose death was occasioned by an enormous fibro-nuclear tumour growing from the abdominal parietes, and general anasarca.

The existence of starch was determined in the liver, spleen, supra-renal capsules, and pancreas; in the last mentioned organ the rarer variety. It was found in all parts of the tumour with the greatest facility.

*Obs.* 9 and 10. A female, E. G., æt. 16 years, and a man, A. C., 43 years of age, both of whom died of phthisis.

The following organs, which appeared healthy in both, contained the ordinary quantity :—the livers, kidneys, pancreatic and mesenteric glands. The supra-renal capsules of A. C. were examined, and with a like result.

*Obs.* 11. A man, æt. 33 years, who died of jaundice, together with œdema and erysipelas of the lower extremities.

All the organs mentioned in the two preceding cases were examined, and positive evidence of the presence of starch obtained ; but the quantity in each was excessively small, more especially in the pancreas. With the exception of the liver, which was in an incipient state of cirrhosis, and deeply impregnated with the coloring matter of the bile, all the viscera were normal.

Among, and also within, the wavy bundles of fibrous tissue from the mesentery, starch grains of the ordinary dimensions were scattered with tolerable regularity, but for the most part at rather wide intervals.

*Obs.* 12. A child three weeks old. Cause of death unknown.

The liver contained a more than ordinary quantity : the kidney, spleen, and pancreas, the usual amount.

*Obs.* 13. A man, D. L., æt. 25 years, who died of diabetes mellitus.

Starch was of excessively rare occurrence in the liver of this subject; less rare in the pancreas; and the general amount observed in the spleen, kidneys, mesentery, and the white and grey matters of the cerebrum. Little was seen in the cerebellum.

In the ovaries and healthy portions of the lungs of two individuals who died of phthisis, its presence was determined; but none was discovered in the muscular substance of their hearts.

With a view of deciding whether starch is peculiar to the human subject, I submitted to examination the cerebrum, cerebellum, the liver, lung, spleen, kidney, striped muscular and areolar tissues, from the sheep ; also the liver, lung, and yellow elastic tissue from the ligamentum nuchæ of the ox, the same facts being established regarding the structures from these animals as were previously mentioned with respect to man.

My observations have not been restricted to organs and tissues entirely, but extended to the various secretions and products of the body, in their normal as well as abnormal conditions. Thus, I have seen starch in healthy blood drawn from the finger, in pus, cancer juice, chronic lymph, bronchitic sputum, icthyosis scales, tubercular matter from the lung, and in the urine (both alkaline and acid) of patients laboring under divers maladies :—Bright's disease, dyspepsia, acute articular rheumatism, pneumonia, etc. In some instances, it was associated with oxalates and phosphates, in others with uric acid and urates, and, again, as in the case of Bright's disease, and of a convalescent from rheumatism, with no deposit, save an almost inappreciable amount of epithelial debris.*

* The great majority of the above cases have occurred in the clinical wards of Prof. Bennett, to whom I have shown these bodies in the tissues and fluids referred to, and who has confirmed their presence.

It may appear astonishing or perhaps incredible to many, that a substance so universally distributed should for such a long period have remained almost unnoticed by microscopical observers. The solution of this apparent enigma will, however, appear sufficiently simple when fully explained. It is well known to those who have applied themselves to the histological study of the various organs and tissues that fatty matters, frequently in the form of oil-globules, are almost invariably interspersed among the morphological elements of which they are composed. Now, to these globules the animal starch corpuscle bears a very marked resemblance, not merely in general form, but likewise in refractile properties. Can it then be looked upon as a matter of surprise, that the two have been confounded by those who have neither by chemical nor other means endeavored to discover their nature? In some instances, when recognised, as they often have been in the urine, they were considered either as fraudulent additions to, or accidental admixtures with, this excretion; possibly, with correctness in some cases, though by no means in all, as I have satisfied myself by experiments the most rigid.

Professor Bennett has pointed out to me the observations made by Schrant on Colloid corpuscles, and from the figures published by that observer, it would appear very probable that these also must occasionally have been mistaken for those of starch.*

If, then, the facts here recorded be taken for granted, the question immediately suggests itself:—are starch granules, when met with in the localities enumerated, the offspring of pathological causes, or the results of the healthy action of the economy? The majority of cases cited clearly show that they must generally be esteemed as physiological products, although, as with the other constitutents of the healthy body, an excess or diminution of them, or an *error loci*, may induce or constitute disease, and will probably hereafter be found to do so.

Regarding their functions, little can in the present state of our knowledge be said, except, perhaps, that they may be looked upon as thermogenic magazines, analogous to, but not identical in action with, the fat cell. We may indeed suppose, from their easy convertibility into grape sugar, and of this by oxidation into carbonic acid and water, that from them caloric is generated when a sudden demand is made for it by the system. Another, and a totally different view may be taken of the purpose they subserve in the economy, and one which does not carry us beyond the limits of possibility; viz., that instead of being converted into the compounds previously mentioned, they are simply metamorphosed into the lactic acid of the gastric secretion.

I certainly anticipated that an examination of the organs from the diabetic subject would have furnished a clue to the comprehension of the physiological and pathological importance of starch, but in vain. Upon reference to this case, it will be observed that, although of very rare occurrence in the hepatic and pancreatic organs, the usual amount was present in the others, so that no conclusive inferences could be drawn.

The development of individual starch grains in the plant is involved

**De Colloid-metamorphose der cel.*

in so much obscurity that botanists have but partially elucidated the subject. I therefore refrain from hazarding an opinion upon their mode of growth in the animal tissues, though inclined to believe that both in the plant and animal their development is similar. In the former, as a general rule, a number of corpuscles is produced within the cavity of a cell; in the latter, this condition is apparently the exception. I have certainly, now and then, seen them occupying the cavity of large flaccid cells, but more frequently either isolated, or aggregated and cohering by interposed homogenous matter, which was either tinged yellow or remained unaffected by the solution of iodine.

Before concluding, let me remark that the chief aim in bringing these crude facts before the public has been to show that man, in common with some of the lower animals, must be regarded as a starch secreting organism, thus adding strength to that connection which exists between the animal and vegetable worlds.—*Edinburgh Medical Journal.*

